# Vascular channels in metacarpophalangeal joints: a comparative histologic and high-resolution imaging study

**DOI:** 10.1038/s41598-017-09363-2

**Published:** 2017-08-21

**Authors:** A. Scharmga, K. K. Keller, M. Peters, A. van Tubergen, J. P. van den Bergh, B. van Rietbergen, R. Weijers, D. Loeffen, E. M. Hauge, P. Geusens

**Affiliations:** 1 Department of Medicine, division of Rheumatology, Maastricht University Medical Centre, Maastricht, The Netherlands; 2 NUTRIM School of Nutrition and Translational Research in Metabolism, Maastricht University, Maastricht, The Netherlands; 3 CAPHRI Care and Public Health Research Institute, Maastricht University, Maastricht, The Netherlands; 4 Department of Rheumatology, Aarhus University Hospital, Aarhus, Denmark; 5 Faculty of Medicine and Life Sciences, Hasselt University, Hasselt, Belgium; 6 Department of Internal Medicine, Viecuri Medical Center, Venlo, The Netherlands; 7 Department of Biomedical Engineering, Eindhoven University of Technology, Eindhoven, The Netherlands; 8 Department of Radiology, Maastricht University Medical Center, Maastricht, The Netherlands; 9 Department of Clinical Medicine, Aarhus University, Aarhus, Denmark

## Abstract

We evaluated whether cortical interruptions classified as vascular channel (VC) on high-resolution peripheral quantitative computed tomography (HR-pQCT) could be confirmed by histology. We subsequently evaluated the image characteristics of histologically identified VCs on matched single and multiplane HR-pQCT images. Four 3-mm thick portions in three anatomic metacarpophalangeal joint specimens were selected for histologic sectioning. First, VCs identified with HR-pQCT were examined for confirmation on histology. Second and independently, VCs identified by histology were matched to single and multiplane HR-pQCT images to assess for presence of cortical interruptions. Only one out of five cortical interruptions suggestive for VC on HR-pQCT could be confirmed on histology. In contrast, 52 VCs were identified by histology of which 39 (75%) could be classified as cortical interruption or periosteal excavation on matched single HR-pQCT slices. On multiplane HR-pQCT images, 11 (21%) showed a cortical interruption in at least two consecutive slices in two planes, 36 (69%) in at least one slice in two planes and five (10%) showed no cortical interruption. Substantially more VCs were present in histology sections than initially suggested by HR-pQCT. The small size and heterogeneous presentation, limit the identification as VC on HR-pQCT.

## Introduction

Erosions are the hallmark of bone destruction in rheumatoid arthritis (RA)^1^. They present as cortical interruptions on conventional radiography (CR), magnetic resonance imaging and ultrasound of the joints, and are the result of inflammatory tissue that stimulates osteoclastic bone resorption^1, 2^. Another imaging technique is High-Resolution peripheral Quantitative Computed Tomography (HR-pQCT), which has a higher sensitivity than the gold standard CR to detect cortical interruptions in finger joints^3–5^. Using HR-pQCT, cortical interruptions have been found in healthy subjects, some of which are hypothesized to be vascular channels (VCs)^1, 6^. Under normal conditions, the only documented peri-articular cortical interruptions in human anatomic specimens are blood vessels that perforate bone at the epiphysis^7^ and at the enthesis^8^. Vascular connections between bone marrow and the joint are an anatomical location for bone damage in animal models of arthritis^8, 9^ as they provide direct entry points for synovitis and osteoclast-mediated joint destruction^9, 10^. In addition, angiogenesis, the *de novo* capillary outgrowth from pre-existing blood vessels, is a characteristic finding in RA and one of the initial features of the inflammatory response that facilitates migration of inflammatory cells^11^. It has therefore been hypothesized that bone damage in RA might start in VCs^1, 9, 10^. However, the actual presence of VCs in the finger joints has not been investigated in human subjects. We have previously shown that a heterogeneous pattern of cortical interruptions in finger joints can be seen by HR-pQCT^6^. The Study grouP for xtrEme Computed Tomography in Rheumatoid Arthritis (SPECTRA) collaboration has formulated a preliminary definition for VCs when using HR-pQCT imaging^12^. Using HR-pQCT, Boutroy *et al*. studied the SPECTRA definition for VCs in a barium perfusion study of an MCP joint and reported that two VCs detected on HR-pQCT, were indeed a VC^13^. However, it is unknown whether this proposed definition identifies VCs correctly and captures all VCs in an MCP joint.

The aim of this study was twofold: First, to study in MCP joints whether the cortical interruptions classified as VCs on HR-pQCT using the SPECTRA definition, are indeed VCs on histology sections. Second, to evaluate the image characteristics of histologically identified VCs on matched single slices of HR-pQCT images and on matched multiplane HR-pQCT images.

## Methods

### Specimens

Anatomic specimens of ten human fingers, each right hand index fingers of ten different individuals, were obtained from the Department of Anatomy and Embryology of the University of Amsterdam, Amsterdam, the Netherlands. The donors had dedicated their body by testament signed during life to the Department of Anatomy and Embryology of the University of Amsterdam, the Netherlands. A handwritten and signed codicil from each donor, posed when still alive and well, is kept at the Department of Anatomy and Embryology, University of Amsterdam, Amsterdam, the Netherlands. The medical history of the donors was unknown.

### HR-pQCT image acquisition

HR-pQCT (XtremeCT1, Scanco Medical AG, Switzerland) scans were performed at clinical *in vivo* settings, i.e. at 60 kVp tube voltage, 900μA tube current, 100 ms integration time, and with a voxel size of 82 μm^6^. The total region scanned was 144 mm (1760 slices) per finger from top distal phalangeal joint to proximal to the metacarpal head (Supplementary Figure 1). The total volumetric bone mineral density (vBMD) of the MCP joint (length in axial direction 18.04 mm) was calculated using standard evaluation protocol of the manufacturer^14^. Scans of HR-pQCT were exported in Digital Imaging and Communications in Medicine (DICOM) format and analyzed using the Osirix (v.5.8.5 64-bit) multiplanar DICOM viewer, viewed on an iMAC 27 inch computer with 2560 × 1440 pixels.

### Selection of regions in MCP joints based on HR-pQCT images

**Figure 1:**
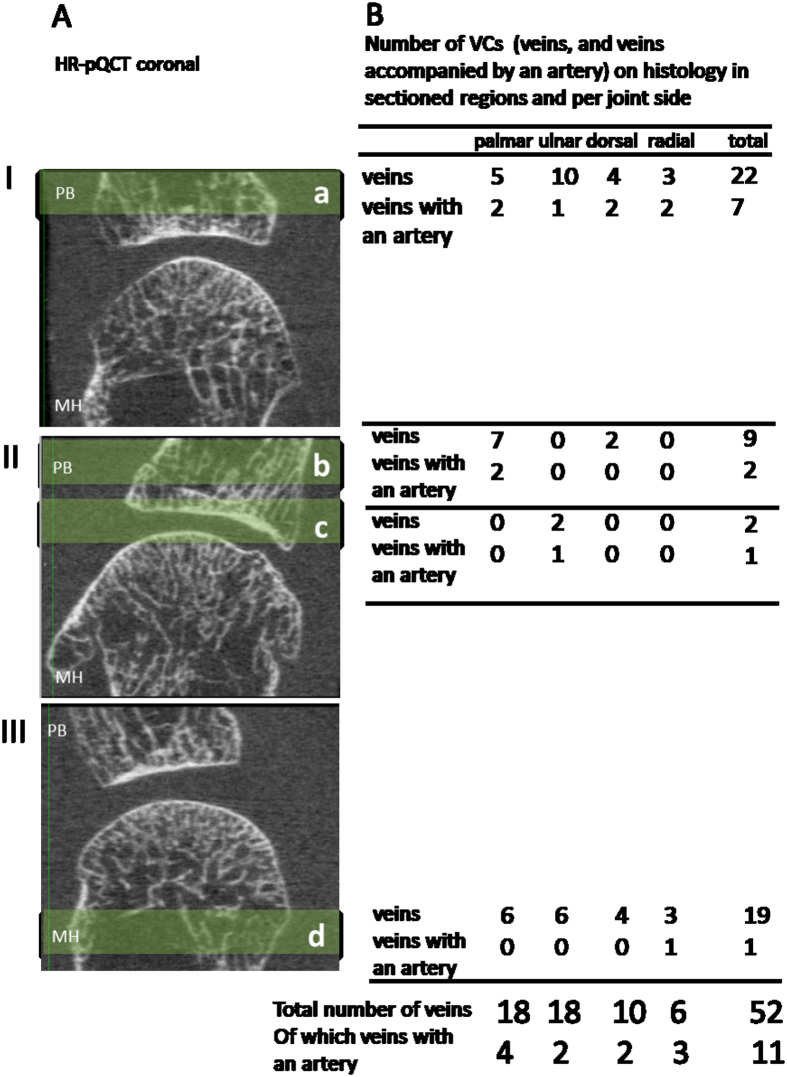
Evaluated regions and distribution of vascular channels. Panel I and II show four regions sectioned (a, b, and c) in the phalangeal base. Region c contained subchondral bone. Panel III shows a region sectioned (d) proximal to the MCP joint. The table (B) shows the distribution of vascular channels (veins, including veins accompanied by an artery) on HR-pQCT and the distribution on palmar, ulnar, dorsal and radial sides. Abbreviations: MCP; metacarpophalangeal, HR-pQCT; High Resolution peripheral Quantitative Computed Tomography, PB; phalangeal base, MH; metacarpal head, VCs; vascular channels.

Prior to histologic examination, two readers independently scored the HR-pQCT images^6^. One reader scored the images twice. The ten MCP joints were scored for the presence of cortical interruptions in at least two slices in two orthogonal planes (further referred as 2 × 2 slices). These cortical interruptions were, according to the SPECTRA collaboration, suspected to be a VC when a cortical interruption was characterized by a parallel cortical lining, present on at least 2 × 2 slices (Supplementary Figure 2)^13, 15^. Based on the presence (kappa, κ) and the total number of cortical interruptions (intraclass correlation coefficient, ICC), intra-reader reliability was moderate to substantial (range 0.52–0.75). Inter-reader reliability for the presence and total number of cortical interruptions was fair to moderate (range 0.37–0.55)^6^. Subsequently, four peri-articular regions in three MCP joints (average 3.4 mm) with the highest number of cortical interruptions were selected for histological sectioning (Fig. 1).

### Histology preparation and histology image analysis

The four selected regions in MCP joints were fixed in formalin followed by dehydration in alcohol, propanol and finally xylene. Subsequently the joint was infiltrated with methylmethacrylate and embedded undecalcified in methylmethacrylate for sectioning. Fourteen-µm-thick sections were cut, transversal to the length of the finger, on a microtome (Reichert Jung GmbH, Heidelberg, Germany). Every second section was stained with Masson-Goldner trichrome, resulting in 478 histology sections in total. Subsequently all the sections were analyzed with a microscope (Nikon ECLIPSE 80i, Tokyo, Japan) equipped with a motorized Proscan 11 stage (Prior, Cambridge, UK), a MT1201 microcator (Heidenhain, Traunreit, Germany) and a DP72 digital camera (Olympus, Tokyo, Japan). The microscope system was connected to a PC with the stereological software newCAST which was used to visualize the sections on the PC screen (version 4.2.1.0, Visiopharm, Hørsholm, Denmark). A total magnification of 23 was used to visualize cortical interruptions and subsequently a magnification of 230 to visualize VCs.

First, cortical interruptions that fulfilled the SPECTRA definition for a VC on HR-pQCT were examined for confirmation on histology. Second, and independently of HR-pQCT results, a histologist systematically analyzed the 478 histology sections for the presence of cortical interruptions. Subsequently, a cortical interruption was defined as a VC when it contained an artery and/or a vein. The maximum width of a histologically identified VC was measured using standard histology measurement methods.

### Image analysis of histologically identified VCs on matched single HR-pQCT slices

The transverse histology sections, in which a VC was identified, were visually matched and compared to corresponding single transverse HR-pQCT slices. Matching was based on the location and similarities of the surrounding normal bone structures and on the distance within the selected regions. All images were analyzed by one reader for the identification of a full cortical interruption or a periosteal excavation without full interruption of the cortex.

### Image analysis of histologically identified VCs on matched multiplane HR-pQCT images

In addition to the matched single slice examination, histologically identified VCs were also compared to matched multiplane HR-pQCT images by two readers in order to specify the image characteristics of histologically identified VCs in terms of the presence of a cortical interruption in at least two consecutive slices in two planes (2 × 2) or less (1 × 2) and whether they fulfilled the SPECTRA definition of a VC^13, 15^.

### Statistical analysis

Descriptive statistics and frequencies were used to calculate the mean with SD for continuous data and the number of VCs. Independent samples t-test was used to compare width measured on histology between VCs containing veins only or both veins and arteries. Analyses were performed with SPSS Statistics for Windows version 23.0 (IBM Corp., Armonk, NY).

## Results

The mean age of the specimen’s subjects was 84.7 (SD 5.5) years. Regions selected for histology are shown in Fig. 1. The average total vBMD of the three MCP joints was 219.7 mgHA/cm^3^.

In the selected regions of the MCP joints, seven cortical interruptions suggestive for a VC according to the SPECTRA criteria were detected on HR-pQCT. When comparing these images to histology, two were not evaluable on histology (due to poor quality of the histology section), in four cases no cortical interruptions could be found on histology and only one was found to be a VC on histology (Supplementary Figure 3).

In contrast, in the 478 14-µm-thick sections that were examined histologically, 52 VCs were identified, and five cortical interruptions without any veins or arteries. All 52 VCs contained a vein, of which 11 also an artery. The veins were located at the ulnar (n = 18), radial (n = 6), dorsal (n = 10) and palmar sides (n = 18) (Fig. 1). Accompanying arteries were located at the palmar (n = 4), ulnar (n = 2), dorsal (n = 2) and radial (n = 3) sides. The width of VCs on histology ranged from 0.049 to 0.790 mm (mean 0.273 mm, SD 0.177 mm). The mean width of histologically identified VCs was significantly larger in VCs containing both veins and arteries (mean width 0.408 mm, range 0.146–0.790 mm) than those containing veins only (mean width 0.236 mm, range: 0.049–0.670 mm, *p* < 0.05). Six histologically identified VCs (11.5%) were smaller than the HR-pQCT voxel size of 82 μm.

**Figure 2:**
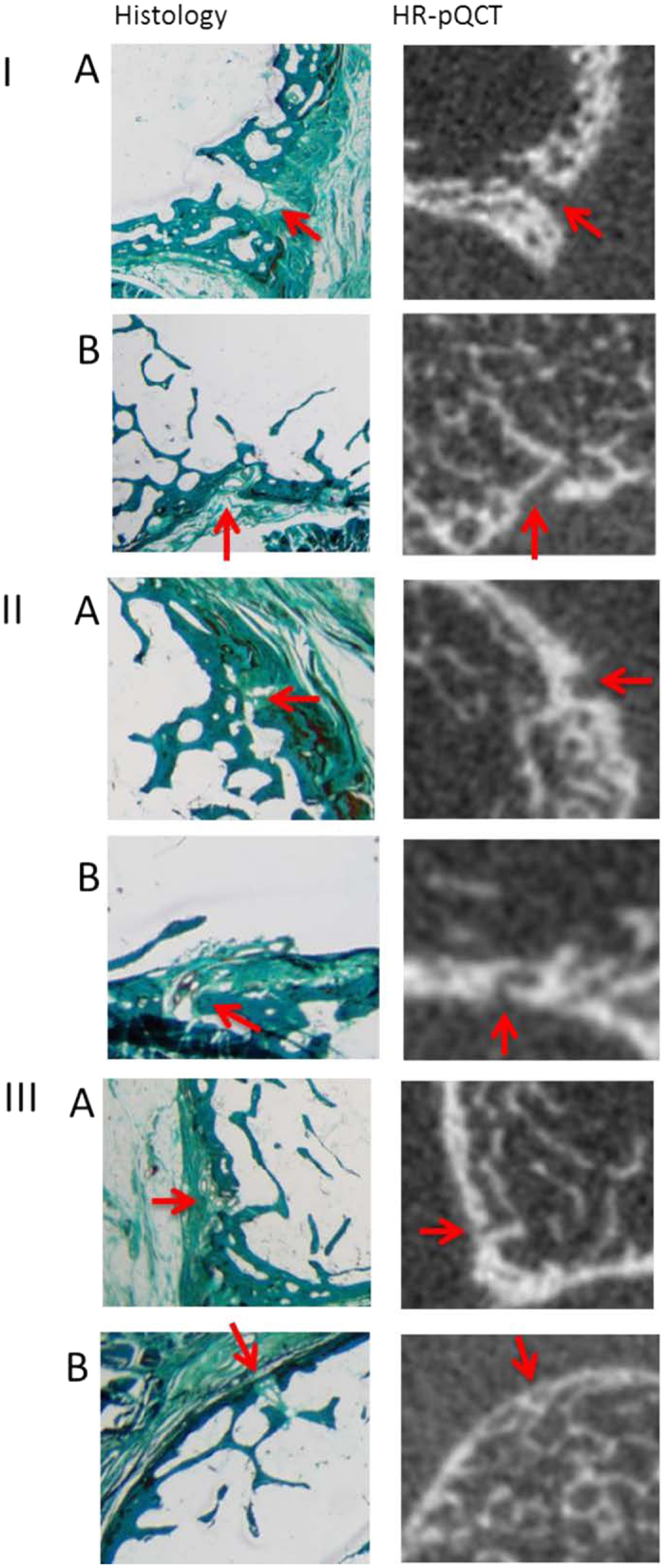
Histology sections and HR-pQCT slices of vascular channels. Panel I Histologically identified vascular channels with cortical interruptions on HR-pQCT slices with full cortical interruptions, that penetrated the cortex in a straight (A) or oblique (B) direction on HR-pQCT. Panel II Histologically identified vascular channels with a clear periosteal excavation without complete interruptions of the cortex on HR-pQCT, being simple excavations (A), or complex in configuration, following the intracortical curvature of the vessel (B). Panel III Histologically identified vascular channels with no clear cortical interruptions on HR-pQCT.

All histology sections containing a VC could be visually matched to corresponding single transverse HR-pQCT slices. In 25 (48.1%) of the histologically identified VCs, single matched HR-pQCT slices showed full cortical interruptions that penetrated the cortex in a straight (Fig. 2 Panel I a) or oblique direction (Fig. 2 Panel I b). In 14 (26.9%) of the histologic identified VCs, single matched HR-pQCT slices showed a clear periosteal excavation without complete interruption of the cortex. These were identified as simple excavations, reflecting periosteal entrance of the vessel (Fig. 2 Panel II a), or complex in configuration, following the intra-cortical curvature of the vessel (Fig. 2 Panel II b). In 13 (25%) of the histologically identified VCs, no clear cortical interruption or excavation could be identified on single matched HR-pQCT slices (Fig. 2 Panel III a and b).

**Figure 3:**
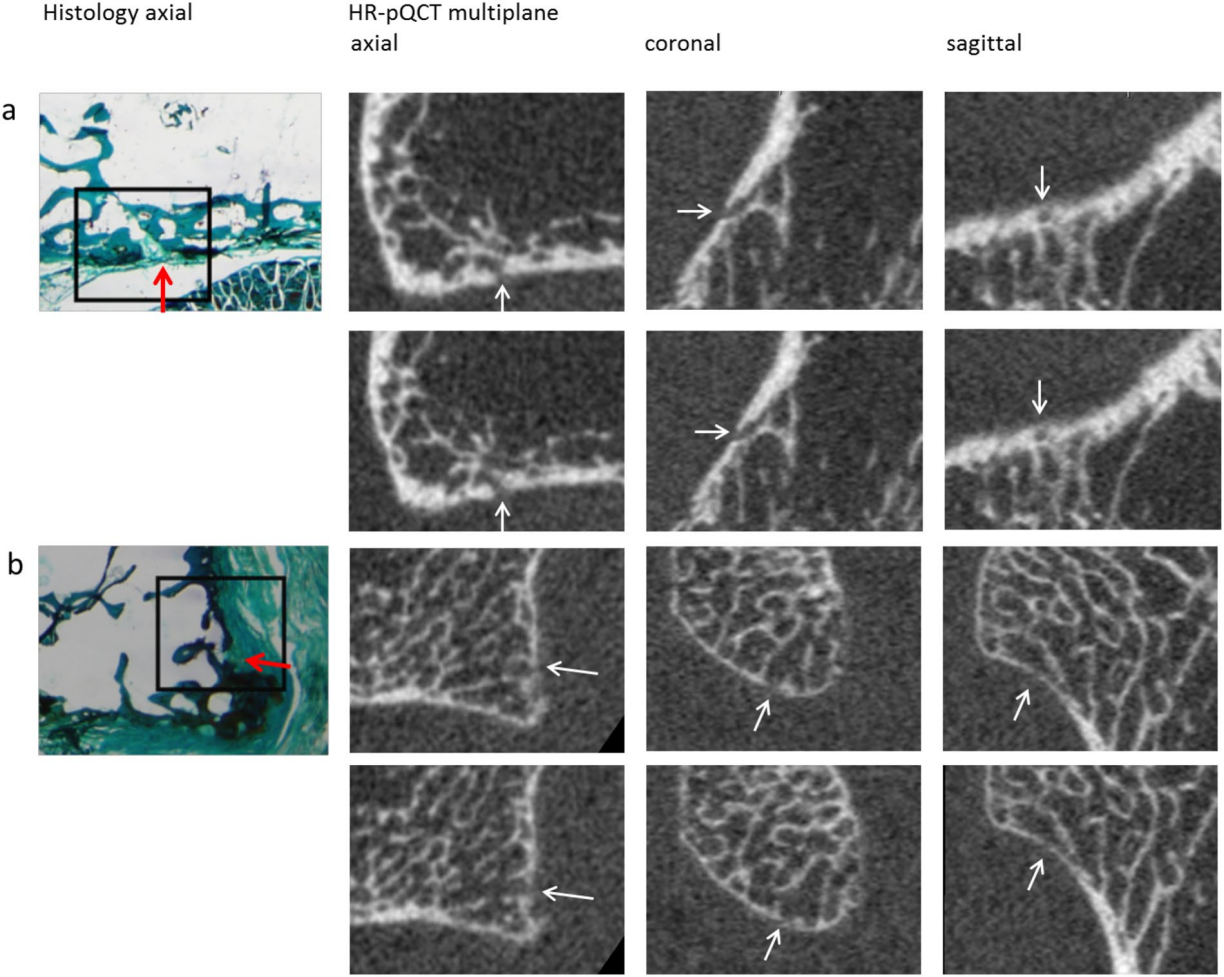
Examples of vascular channels identified on histology with matched multiplane HR-pQCT images of a vascular channel (panel a), and an interruption in the cortex not defined as a vascular channel according to SPECTRA (panel b).

The 52 histology sections with a VC were further compared to matched multiplane HR-pQCT images. Eleven (21%) of the histologically identified VCs showed cortical interruptions in at least 2 × 2 slices, 36 (69%) in at least 1 × 2 slices and no cortical interruption in five (10%). Five of the 11 cortical interruptions on 2 × 2 slices and 15 of the 36 cortical interruptions on 1 × 2 slices had a parallel cortical bone lining on HR-pQCT. Figure 3 shows two examples of matched histology slices to HR-pQCT images.

## Discussion

In the selected periarticular regions of MCP joints of anatomic specimens, substantially more VCs were identified on histology than initially expected from HR-pQCT images^13^. These histologically identified VCs were heterogeneous and many also small, making them difficult to identify on HR-pQCT. The finding of 52 VCs on histology was unexpected, as the initial analyses of HR-pQCT images based on the SPECTRA criteria only suggested the presence of seven VCs and an earlier perfusion study only found two VCs^13^. This study is the first to formally indicate the ample presence of histologically identified VCs in MCP joints. Our findings have consequences for the interpretation of HR-pQCT images and can be of clinical significance.

First, the VCs were heterogeneous in configuration on HR-pQCT. VCs presented as full cortical interruptions (straight or oblique penetration of the cortex) or excavations (simple or complex configuration). The heterogeneity of VC images on HR-pQCT is in line with earlier findings that reported variable image characteristics of cortical interruptions on HR-pQCT^3, 16^. By using predefined image characteristics of VCs according to SPECTRA, seven cortical interruptions fulfilled the SPECTRA definition of a VC (i.e. parallel cortical lining on two consecutive slices in two planes) on HR-pQCT, five could be evaluated on histology and only one was a true VC on histology. Therefore, the current definition of a VC on HR-pQCT imaging appears insufficient to correctly identify VCs. The heterogeneity of the VCs on HR-pQCT needs to be incorporated in the development of a new definition.

Second, the histologically identified VCs were small in size with a mean width of 0.273 mm. While in a barium perfusion experiment using HR-pQCT only two VCs were identified, such perfusion studies are presumably insufficient to detect small VCs^13^. In this study we used histologically identified VCs and matched them to their corresponding location on HR-pQCT images. In 13 (25%) of the histologically identified VCs, no clear cortical interruption or excavation could be identified on single matched HR-pQCT slices and in 5 (10%) of the histologically identified VCs, no cortical interruption was seen on multiplane HR-pQCT images. On histology, six identified VCs (11.5%) were smaller than the HR-pQCT voxel size of 82 μm. The small size of the VCs and voxel size of HR-pQCT explains the observation that multiple VCs could not be identified as cortical interruptions on HR-pQCT. The smallest details that can be seen on the HR-pQCT scans are 82 μm (the voxel size). Cortical interruptions smaller than this size are only partly covered by a voxel (‘partial volumes’) and may not be detectable. On the other hand, very thin cortices may be identified as an interruption on HR-pQCT scanning. To investigate the potential errors involved due to these partial volume effects, we compared in an earlier study the detection of cortical interruptions on HR-pQCT imaging with that on micro computed tomography images with a much higher resolution (18 μm voxel size)^6^. In that study, we found that the sensitivity of HR-pQCT for detecting cortical interruptions was 81.6% (positive predictive value was also 81.6%) while the specificity was 64.0%. No sensitivity can be calculated from the comparison with histology since the evaluations on HR-pQCT and histology were independently performed with different aims for each modality. For clinical application, the previously obtained sensitivity and specificity values were considered acceptable. When developing a new definition, for example including cortical interruptions on less than 2 × 2 slices, the small size and voxel size of HR-pQCT should be taken into consideration. Visual interpretation of HR-pQCT images is a complex task, as has been shown in studies that evaluated trabecular bone structure and the lining of the endocortical bone^17^. In the field of metabolic bone diseases, this has been solved by fully or semi-automated image analysis algorithms. We are currently developing computer-assisted semi-automatic and fully automatic algorithms that might facilitate evaluation of the presence, size and location of cortical interruptions and VCs.

Third, the VCs were spread around the joint, which is the main location for inflammation in RA. It has previously been shown in animal models of arthritis and hypothesized in humans, that vascular connections can play a pathophysiologic role in the formation of erosions when joint inflammation occurs^9, 10, 18^. Two different scenarios have been proposed for the pathogenesis of peri-articular erosions in RA; the inside-out and outside-in scenarios^19^. In the conventional view, the outside-in concept hypothesizes that RA starts with inflammation in the synovial membrane, spreading locally to adjacent structures including the bone marrow through cortical interruptions^19^. The high number of histologically identified peri-articular VCs supports that inflammation can indeed spread locally through VCs to the bone marrow from these joint locations. The alternative view is the inside-out scenario, which hypothesizes that inflammation starts in the bone marrow and migrates locally through peri-articular cortical interruptions into the joint^19^. VCs facilitate local homing of osteoclast precursor cells, which upon contact with the appropriate molecular signals such as anti-citrullinated protein antibody (ACPA), can differentiate into osteoclasts in the preclinical phase of RA^1^. The high number of VCs around MCP joints in our study could explain why for example ACPA positive subjects develop peri-articular erosions before the occurrence of synovitis.

This study has several limitations. First, the study was conducted in a sample of anatomic specimens of elderly women with unknown medical background, preserved in formalin, and with low mineralized bone. The relatively old age might have affected the condition and visual observations on HR-pQCT imaging of the fingers. Average vBMD in the specimens was 219.7 mgHA/cm^3^, which is lower than observed in the normal population (>300 mgHA/cm^3^)^5, 20^. Second, only MCP joints were studied and the results might therefore not be applicable to other joints. However, we deliberately selected MCP joints, as these joint locations are frequently the first to be involved in the development of RA and where erosions might occur even before RA becomes clinical^1^.

The strength of this study is that imaging by histology and HR-pQCT was performed independently.

To conclude, substantially more VCs were present in histology sections than initially suggested by HR-pQCT. Although most histologically identified VCs could be identified as cortical interruptions in matched HR-pQCT slices, their small size and heterogeneous presentation, limit the identification as VC on HR-pQCT images. New definitions for VCs on HR-pQCT should take this into consideration.

## Key Messages

- Substantially more VCs were present in histology sections than initially suggested by HR-pQCT.
- The preliminary SPECTRA definition is insufficient to capture most VCs using HR-pQCT.
- The heterogeneous presentation of VCs has to be taken into account when interpreting HR-pQCT images.

### Availability of data and Materials

The datasets generated during and/or analyzed during the current study are available from the corresponding author on reasonable request.

## Electronic supplementary material

Supplementary Information
