# Supplementary material for: Vascular channels in metacarpophalangeal joints: a comparative histologic and high-resolution imaging study
Source: Sci Rep. 2017 Aug 21;7:8966. doi: 10.1038/s41598-017-09363-2 (PMC5566954; doi:10.1038/s41598-017-09363-2)
Supplement: Supplementary file 1 — Supplementary Information [file 41598_2017_9363_MOESM1_ESM.pdf]

Vascular channels in metacarpophalangeal joints: a comparative histologic and high-resolution imaging study

A. Scharmga*[1,2,3], K.K. Keller*[4], M. Peters[1,2,3], A. van Tubergen[1,3], J.P. van den Bergh[1,2,5,6], B. van Rietbergen[7], R. Weijers[8], D. Loeffen[8], E.M. Hauge[4,9], P. Geusens[1,3,5]

[1]Department of Medicine, division of Rheumatology, Maastricht University Medical Centre, Maastricht, the Netherlands
[2]NUTRIM School of Nutrition and Translational Research in Metabolism, Maastricht University, Maastricht, the Netherlands
[3]CAPHRI Care and Public Health Research Institute, Maastricht University, Maastricht, the Netherlands
[4]Department of Rheumatology, Aarhus University Hospital, Aarhus, Denmark
[5]Faculty of Medicine and Life Sciences, Hasselt University, Hasselt, Belgium
[6]Department of Internal Medicine, Viecuri Medical Center, Venlo, the Netherlands
[7]Department of Biomedical Engineering, Eindhoven University of Technology, Eindhoven, the Netherlands
[8]Department of Radiology, MUMC+, Maastricht, the Netherlands
[9]Department of Clinical Medicine, Aarhus University, Aarhus, Denmark

*AS and KK contributed equally to this paper.

SUPPLEMENTARY FILES (3 Figures)

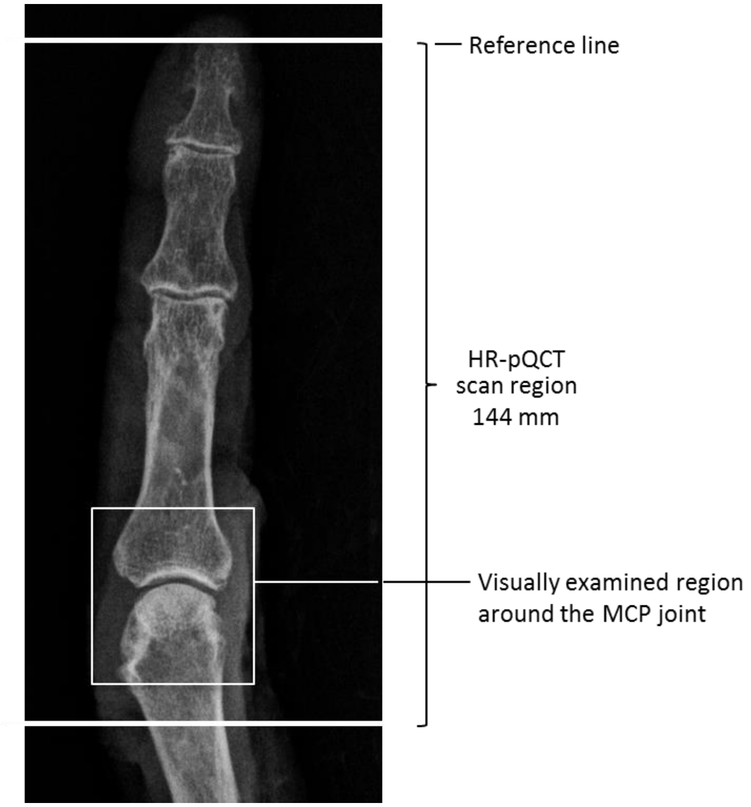

Supplementary Figure 1.
Scan region of index finger and visually examined region on HR-pQCT

Abbreviations: HR-pQCT; High Resolution peripheral Quantitative Computed Tomography

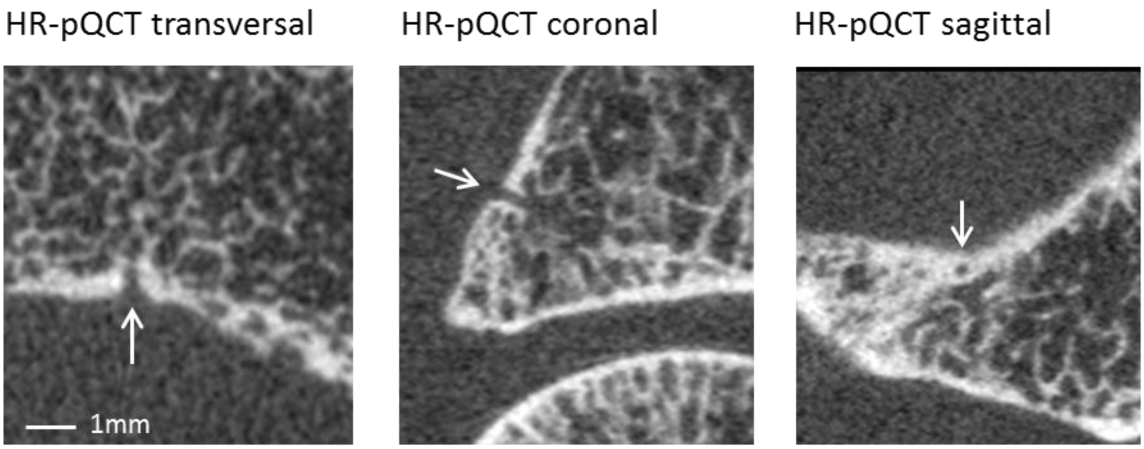

Supplementary Figure 2.
Example of a cortical interruption in the MCP joint in transversal, coronal and sagittal slices that was classified as a vascular channel according to the SPECTRA definition, based on having a parallel structure (arrow).

Abbreviations: MCP; metacarpophalangeal, HR-pQCT; High Resolution peripheral Quantitative Computed Tomography, SPECTRA; The Study grouP for xtrEme Computed Tomography in Rheumatoid Arthritis

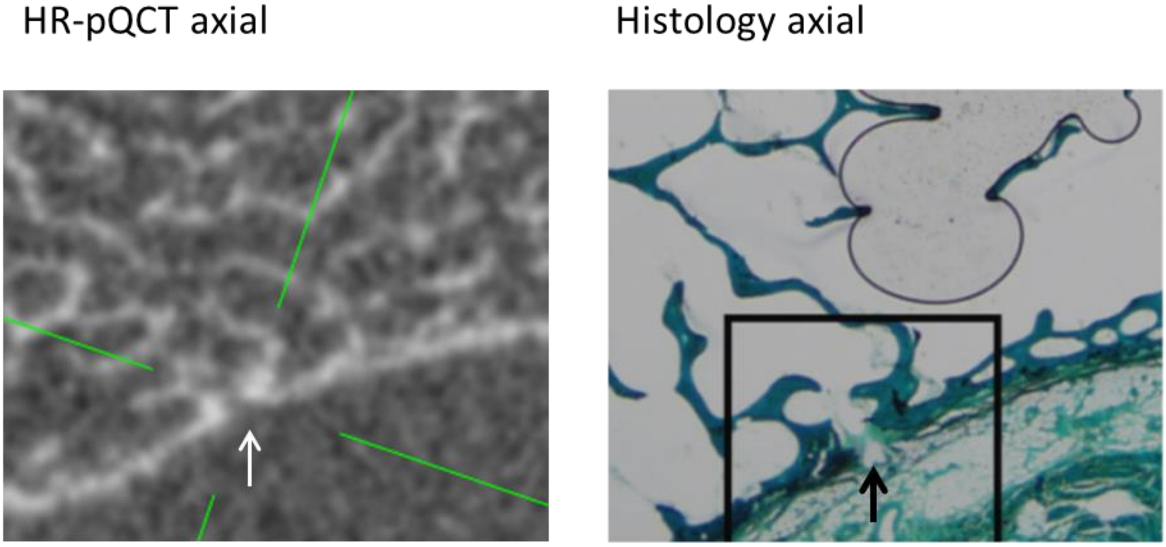

Supplementary Figure 3.
Example of a VC as defined on HR-pQCT with confirmation on histology.
An interruption defined VC (arrow) by HR-pQCT (left) and confirmed as VC (arrow) on histology (right)

Abbreviations: HR-pQCT; High Resolution peripheral Quantitative Computed Tomography, VC; vascular channel
